# Comparing general obesity indicator and central obesity indicators for hypertension prediction

**DOI:** 10.6026/973206300213735

**Published:** 2025-10-31

**Authors:** Divyangkumar N Patel, Nilesh G Patel, Mehul R Patel

**Affiliations:** 1 Department of Community Medicine, Dr N. D. Desai Faculty of Medical Science and Research, Dharmsinh Desai University, Nadiad, Gujarat, India; 2 Department of Community Medicine, Smt B. K Shah Medical Institute and Research Center, Sumandeep Vidyapeeth deemed to be university, Gujarat, India; 3 Department of Community Medicine, Nootan Medical College and Research Center, Sankalchand Patel University, Gujarat, India

**Keywords:** Receiver's operator characteristic (ROC) curve, hypertension, BMI, central obesity

## Abstract

Hypertension has emerged as a global epidemic, particularly affecting developing countries like India. Central obesity, assessed through waist circumference (WC) and waist-to-hip ratio (WHR), is closely linked to cardio-metabolic disorders such as hypertension, cardiovascular disease, metabolic syndrome and diabetes mellitus. Therefore, it is of interest to assess the predictive capability of anthropometric indicators and identifying individuals at risk of high blood pressure. A cross-sectional study was conducted among Bhavnagar police personnel, involving measurements of both central and general obesity through anthropometric assessments. Thus, we show that BMI and waist circumference which indicating general and central obesity respectively, both are associated with hypertension with marginal superiority of BMI.

## Background:

India is witnessing a swift escalation in the prevalence of non-communicable diseases, notwithstanding its substantial burden of infectious diseases and maternal and child health issues [1]. Non-communicable diseases, responsible for 60% of global deaths, include CVD, cancers, respiratory issues and diabetes. CVDs, like heart disease and strokes, cause 17.7 million deaths, with India contributing one-fifth, especially among the younger population [2]. The conventional risk factors like hypertension, diabetes mellitus, dyslipidemia, smoking and obesity are linked to a heightened prevalence of coronary artery disease (CAD) in the Indian population. The escalating burden of these conventional risk factors is contributing to a rapid rise in cardiovascular diseases among young Indians [1, 2]. The Hypertension is already a highly prevalent cardiovascular risk factor worldwide because of increasing longevity and prevalence of contributing factors such as obesity [3]. Hypertension is a modern day's epidemic and it is becoming a public health emergency worldwide, especially in the developing countries like India [4]. Globally around 40% of more than 25 years of age population had high blood pressure [5]. Epidemiological studies have demonstrated that obesity is a significant, independent and modifiable risk factor for hypertension, with numerous research findings indicating a strong correlation between a higher incidence of hypertension and a markedly increased prevalence of obesity [6, 7]. The assessment of overweight and obesity can be accomplished through multiple methods, with simple, widely accepted and direct measures encompassing anthropometric indicators such as BMI, waist circumference and waist-to-hip ratio [8, 9]. Several epidemiological studies consistently highlight the predictive role of various anthropometric measures, such as Body Mass Index (BMI), waist circumference (WC) and waist-hip ratio (WHR), in relation to non-communicable diseases like hypertension, type 2 diabetes mellitus and cardiovascular disease (CVD) [10]. Central obesity most commonly measured by waist circumference (WC) and waist to hip ratio (WHR) is found to be more associated with cardio-metabolic disorder like Hypertension, cardio-vascular disease, metabolic syndrome, Diabetes mellitus etc [11, 12]. It has been recognized that south Asian (subject originating from India, Pakistan, Srilanka, Bangladesh and Nepal) has certain unique clinical and biochemical characteristics that are collectively referred to as the -Asian Indian phenotype. In them despite relatively lower prevalence rates of General obesity as defined by Body mass index, they tend to have larger waist measurement and waist to hip ratios [13]. This study aimed to assess the association between Body Mass Index, Waist to Hip Ratio and Waist Circumference with hypertension (HTN) among the Gujarati population. The research addressed a critical gap in understanding the screening performance of these indicators in predicting HTN in Gujarat, India, where there is a significant burden of HTN and low awareness [14]. Therefore, it is of interest to evaluate the discriminatory capability of BMI, WHR and WC in predicting HTN. The identification of robust predictors can enhance risk assessment protocols, enabling more accurate identification of individuals at an elevated risk of developing hypertension in primary care settings.

## Materials and Methods:

A community based cross sectional study was conducted among police personnel working in Bhavnagar city. Permission was obtained from the ethics committee of Govt. Medical College, Bhavnagar before the start of study. All police personnel of Bhavnagar city of ≥ 30 yrs of age were enrolled for the study. Total 260 out of 294 study subjects were included in the study after applying inclusion and exclusion criteria (Response rate 88 %). After clearance from ethics committee and with prior permission from Police Superintendent of Bhavnagar, data was collected. Inclusion and exclusion criteria were applied to enroll participant in to study. Anthropometric measurements were taken with subjects in light clothing and without shoes. Height and weight were measured using calibrated stadiometer and portable weighing machine respectively. The height and weight were recorded to the nearest centimeters and kilograms respectively. BMI was calculated by dividing weight (kg) by square of height (m2). Waist and hip circumference were measured with a non-stretchable plastic tape. Waist circumference was measured as the minimum horizontal girth between costal margins and iliac crests at the end of normal expiration. The waist circumference was recorded nearest to centimeter. Hip circumference was measured as the maximum circumference of the buttocks over the greater trochanters. The hip circumference was recorded nearest to centimeter. Waist to Hip ratio (WHR) was calculated as Waist circumference (cm) divided by Hip circumference (cm). Blood pressure was measured using JNC VII standard method by a mercury sphygmomanometer with the subject sitting quietly for at least 5 minutes in a chair (rather than on an examination table), with arm supported at heart level. Caffeine, exercise and smoking were avoided for at least 30 minutes prior to measurement. Initially systolic blood pressure was measured using placatory method. The cuff was inflated 20 to 30 mm Hg above this level for the auscultatory determinations; the cuff deflation rate for auscultatory readings was 2 mm Hg per second. Diastolic blood pressure (SBP) was the point at which the first of two or more Korotkoff sounds was heard (onset of phase 1) and the disappearance of Korotkoff sound (onset of phase 5) was used to define diastolic blood pressure (DBP). Two measurements were and the average was recorded. According to JNC VII classification Person was considered hypertensive if his/her systolic blood pressure ≥ 120 mm Hg and/ or diastolic blood pressure ≥ 80 mm Hg or s/he was on hypertensive treatment.

## Statistical analysis:

Data entry was done in Excel 2007 sheet and Statistical analysis was done by Med Calc version 11.6.1.0 (trial version) statistical software. Roc curve was constructed to determine the better predictive power of hypertension among the anthropometric indicators of obesity.

## Results:

Total 260 participants were enrolled into study. Table 1 (see PDF) shows socio demographic profile of study subjects. The majority of study subjects were Hindu 92.31% and 70% of the subjects were from 40-59 years of age. 63.84% subjects belong to nuclear family while, 19.62% and 16.54% subjects residing in third generation and joint family respectively. The prevalence of hypertension among study participant was 18.85%. Figure 1 (see PDF) shows that Area under curve of Receiver Operator Characteristic curve of BMI (0.635) was highest followed by Waist circumference (WC) (0.626) and Waist to hip ratio (WHR) (0.604). The study result shows that, BMI is considered as most important predictor of obesity compared to waist circumference (WC) and Waist to hip ratio (WHR). Hypertension is more associated with general obesity (diagnosed by BMI) compare to central obesity (diagnosed by WC & WHR).

## Discussion:

The World Health Organization has established guidelines for categorizing obesity and overweight status based on Body Mass Index (BMI) and has highlighted the close relationship between BMI and cardiovascular risk factors [12]. Recently, there has been a shift towards using waist circumference and related values as representative indicators of abdominal adiposity due to their correlation with abdominal fat mass and stronger associations with cardiovascular risk factors compared to BMI. However, the effectiveness of these indicators in detecting cardiovascular risk factors has been a subject of controversy [15]. This study indicates that both BMI and waist circumference are equally predicting the presence of hypertension with marginal superiority of BMI over WC in the Indian population. Study conducted by Islam *et al.* showed that BMI was outperformed compared to waist circumference and waist to hip ratio in predicting hypertension in Albanian population [16]. Result found in this study is aligning with result found in our study. A study conducted by Al-Sharbatti *et al.* showing that waist circumference, an indicator of central obesity was the only indicator which showed significant relationship with both systolic and diastolic blood pressure [17]. A study conducted among tribe population of north east India by Maken *et al.* showing that waist circumference having more predicting power compared to body mass index for predicting hypertension [18]. This study was conducted among specific ethnic group (tribe) highlighting that abdominal adiposity indicator has more predicting role compared to general obesity indicator. A study conducted by Beck *et al.* in Brazil showing that the waist-to-height ratio and BMI have shown good areas under the ROC curve; still they suggest the use of waist circumference to predict high blood pressure [19]. Most studies suggest that centrally located body fat is more important determinant of blood pressure elevation than peripheral body fat in both men and women, which is in contrast to our study findings [20]. This difference may be because of different studies conducted among different geographical area among different population having genotypic and phonotypical variation showing waist circumference as the better predictor of hypertension. Our study was conducted among the Gujarati population suggest that the general as well as the abdominal adiposity both are equally associated and predicting hypertension. The study relied on a cross-sectional analysis of obesity indicators as measures of adiposity, preventing the establishment of causal inferences. To address this limitation, future research should employ prospective studies with direct measures of adiposity to enhance the precision of predictive performance among the Indian population. Furthermore, the study focused on police personnel, an occupation group characterized by a higher proportion of males, with only nine female participants. Consequently, the study's generalizability is predominantly applicable to males and may not fully represent females. Despite these limitations, the study boasts key strengths, including a representative sample and the implementation of highly standardized procedures for anthropometric data collection.

## Conclusion:

Our investigation into the predictive relationship between obesity indicators and hypertension underscore the significance of targeted preventive measure. The study concluded that both BMI and WC can behave similarly in their predictive ability for risk assessment of undiagnosed and diagnosed hypertension among adult population. Visceral and general obesity both lead to peripheral vascular resistance, ultimately responsible for development of hypertension. Tailored public health initiatives and clinical interventions can be developed based on these finding to mitigate the risk of hypertension among individuals with obesity.
